# Lung dendritic cells undergo maturation and polarization towards a T helper type 2-stimulating phenotype in a mouse model of asthma: Role of nerve growth factor

**DOI:** 10.3892/etm.2014.1967

**Published:** 2014-09-15

**Authors:** QINGWU QIN, ZHAN WANG, PINHUA PAN, ZU CAO, QING XIA, HONGYI TAN, CHENGPING HU

**Affiliations:** 1 Department of Respiratory Medicine, Xiangya Hospital, Central South University, Changsha, Hunan 410008, P.R. China; 2 Bronchial Asthma Research Center of Hunan Province, Changsha, Hunan 410008, P.R. China

**Keywords:** nerve growth factor, lung dendritic cells, asthma

## Abstract

Nerve growth factor (NGF) and dendritic cells (DCs) have been hypothesized to modulate T cell responses in a mouse model of asthma. However, whether NGF plays a role in regulating the maturation and polarization of lung DCs remains unclear. In the present study, the effect of NGF inhibition on the maturation and phenotype of lung DCs was investigated in a mouse model of asthma. BALB/c mice were sensitized and challenged with ovalbumin (OVA), and subsequently received anti-NGF treatment. At 24 h following the last challenge, airway responsiveness and inflammation were examined. The concentrations of NGF, interferon (IFN)-γ and interleukin (IL)-4 were analyzed. In addition, maturation and CD103 expression in the lung DCs were investigated. Anti-NGF treatment was found to significantly reduce airway hyperreactivity and inflammation in asthmatic mice. In addition, a subdued T helper 2 (Th2) response was observed, characterized by the downregulation of IL-4 and the upregulation of IFN-γ. Furthermore, the expression of the DC surface molecules, CD80, CD86 and major histocompatibility complex class II, as well as the proportion of lung CD103^+^ DCs, decreased in the OVA-sensitized and challenged mice. The proportion of lung CD103^+^ DCs also exhibited a positive correlation with the levels of plasma NGF in the mice. These results may provide an explanation for the role of NGF in amplifying the Th2 response in allergic diseases. Therefore, NGF may promote the maturation and polarization towards a Th2-stimulating phenotype of activated DCs, contributing to an amplification of the Th2 response in asthma.

## Introduction

Allergic asthma affects 1–18% of the population in different countries and therefore, represents one of the most common diseases (1). Asthma is a chronic inflammatory airway disease characterized by recurrent episodes of wheezing, breathlessness, chest tightness and coughing driven by an aberrant T helper 2 (Th2) immune response to environmental allergens (2). In addition to its classical nervous system domain, nerve growth factor (NGF) is regarded as an important factor involved in the pathogenesis of allergic diseases, including asthma (3). Studies in ovalbumin sensitized and challenged asthmatic mice with anti-NGF antibody (4) or small interfering RNA (5) have shown that blocking NGF can improve airway inflammation. In addition, transgenic mice overexpressed NGF in the airways had more severe airway inflammation compared with wild-type mice when ovalbumin sensitized and challenged (4). However, the mechanisms underlying the effects of NGF on inflammation in asthma remain unclear.

Previous studies have hypothesized that NGF functions as an amplifier for Th2 effector functions, playing an important role in the development of airway hyperreactivity (6). Recently, an *in vivo* study demonstrated that anti-NGF treatment inhibited allergic airway inflammation by modulating the balance of pro- and anti-asthmatic T cell responses in the lungs of mice (7). However, the maturation and subtype of lung dendritic cells (DCs) were not investigated in this study. DCs can be found throughout the conducting airways, lung interstitium, vasculature, pleura, and bronchial LN Under basal conditions (8). Serving as innate sensors of foreign antigens/pathogens, DCs recognize microbial patterns, damage induced molecules and cytokines and then migrate to the draining lymph nodes where they activate naive T lymphocytes, affecting the nature of T-lymphocyte differentiation (9). Lambrecht *et al* have shown that in vivo depletion of lung CD11c+ dendritic cells during OVA challenge abolished the characteristic features of asthma, including eosinophilic inflammation, goblet cell hyperplasia, and bronchial hyperreactivity (10). In the absence of CD11c(+) cells, endogenous or adoptively transferred CD4(+) Th2 cells did not produce interleukin (IL)-4, IL-5, and IL-13 in response to OVA aerosol, which were restored by adoptive transfer of CD11c(+) DCs (10). Similarly, They have also demonstrate that inflammatory DCs are necessary and sufficient for induction of Th2 immunity in a house dust mite sensitized and challenged asthmatic mice (11). Collectively, these date suggested that DCs play an important role in the stimulation and polarization of naive T cells towards a Th2 phenotype in the face of allergen exposure. Since NGF can amplify Th2 response as mention above, the present study hypothesized that NGF may be involved in regulating lung DCs maturation and differentiation during the pathogenesis of asthma.

## Materials and methods

### Experimental animals and treatment

Female BALB/c mice (age, 6–7 weeks; weight, 20–22 g) were obtained from the Experimental Animal Center of Central South University (Changsha, China). The mice were housed in a quiet, antigen-free environment, with standardized temperature and humidity and free access to food and water. The experimental protocols were approved by the Institutional Animal Care and Use Committee of Xiangya School of Medicine, Central South University, and were in accordance with the guidelines provided by the National Institutes of Health (Bethesda, MD, USA).

Mice were allowed to acclimatize for one week prior to the start of the experiment, and were then randomly divided into four groups as follows (n=8 for each subgroup): control, asthma, control immunoglobulin G (IgG) and anti-NGF. The mice were treated as previously described (12). Briefly, on days one and eight, all the groups, with the exception of the control group, were sensitized via an intraperitoneal (i.p.) injection of 20 μg OVA (Sigma-Aldrich, St. Louis, MO, USA) and 1 mg aluminum hydroxide hydrate (Sigma-Aldrich) in 500 μl saline. On the same days, the mice in the control group were sham-sensitized with saline. On day 21, all the groups, with the exception of the control group, were challenged once daily for five days with 1% OVA (1% w/v in saline buffer) for 30 min using a nebulizer (Boehringer-Ingelheim, Ingelheim, Germany), while the control mice were exposed to aerosolized sterile saline only. At 30 min prior to the inhalation treatment, mice in the anti-NGF group and control IgG group were injected (i.p.) with an anti-NGF antibody (1:2,000; Millipore Corporation, Billerica, MA, USA) or rabbit IgG (1:2,000; Millipore Corporation) in phosphate-buffered saline (PBS; 4 ml/kg), respectively (13). The control and asthma group received vehicle only.

### Analysis of airway responsiveness

At 24 h following the last OVA challenge, mice were anesthetized and airway responsiveness to methacholine was measured using whole-body plethysmography (PLY 3211; Buxco Electronics, Troy, NY, USA), as previously described (14). Mice were treated for 2 min with aerosolized saline or increasing doses of methacholine generated by an ultrasonic nebulizer, following which airway resistance (RL) was measured. RL was determined by dividing the driving pressure by the rate of air flow. The results for each methacholine concentration were expressed as the percentage of the baseline RL values following 0.9% NaCl exposure. Following the measurement of airway responsiveness, blood samples were collected by cardiac puncture and the mice were sacrificed by exsanguination.

### Lung histology

Samples of the right middle lung lobe were fixed in 4% paraformaldehyde and embedded in paraffin. Tissue sections (4 μm) were stained with hematoxylin and eosin, and morphological changes in the lungs were observed using a light microscope. Peribronchial inflammation of four mice from each group were analyzed and scored as follows: 0, normal; 1, few cells were observed; 2, a ring of inflammatory cells one cell layer deep; 3, a ring of inflammatory cells 2–4 cell layers deep; and 4, a ring of inflammatory cells >4 cell layers deep (15).

### ELISA assay

NGF, interferon (IFN)-γ and interleukin (IL)-4 levels in the serum were quantified using ELISA kits, in accordance with the manufacturer’s instructions (NGF: KA0400; Abnova, Taipei, Taiwan; IFN-γ: DuoSet ELISA DY485; R&D Systems, Minneapolis, MN, USA; IL-4: DuoSet ELISA DY404; R&D Systems). Absorbance was measured at 450 nm using a plate reader.

### Western blot analysis

NGF protein expression levels in the lungs were determined using western blot analysis. Total protein was extracted and 30 μg protein was separated using 8% sodium dodecyl sulfate-polyacrylamide gel electrophoresis. The proteins were electrotransferred to a polyvinylidene fluoride membrane (Millipore Corporation). Non-specific binding was inhibited by incubating the membranes with 0.05 g/ml skim milk powder at room temperature (20°C) for 2 h, following which the membranes were incubated at 4°C overnight with an anti-NGF antibody (1:500; Abcam, Cambridge, MA, USA). Subsequently, the membranes were incubated with a goat anti-rabbit IgG horseradish peroxidase-conjugated antibody (1:2,000; Sigma-Aldrich). Reactions were visualized using enhanced chemiluminescence reagents (Pierce Biotechnology, Inc., Rockford, IL, USA) and quantified using Glyko Bandscan 5.0 software (Glyko, Novato, CA, USA). Results were expressed as the mean band density normalized against β-actin, which was used as an internal control.

### DC isolation and flow cytometric analysis

Single-cell suspensions were prepared from the lungs, as previously described (16). Lung lavage was initially performed using ice-cold PBS with 5 mM EDTA. The lungs were then perfused with 10 ml PBS, containing 10 U/ml heparin, via the right ventricle of the heart until the lungs turned white. The lungs were removed, cut into small fragments and digested with 250 U/ml collagenase D (Roche Diagnostics, Mannheim, Germany). EDTA (final concentration, 10 mM) was added after 5 min to stop the collagenase activity, following which a 100-μm cell strainer (BD Biosciences, Franklin Lakes, NJ, USA) was used to filter the disassociated lungs. Finally, hypotonic lysis was performed to remove the erythrocytes, resulting in a single cell suspension. The CD11c^+^ cells were positively selected using an anti-mouse CD11c magnetic microbeads kit (CD11c N418, catalog no. 130-052-001; Miltenyi Biotec, Bergisch Gladbach, Germany) and autoMACS (Miltenyi Biotec), in accordance with the manufacturer’s instructions (17). Lung DCs were stained using fluorescein isothiocyanate (FITC)-conjugated anti-mouse CD80 (11-0801; eBiosciences, Hatfield, UK), allophycocyanin-conjugated anti-mouse CD86 (17-0862; eBiosciences), phycoerythrin-conjugated anti-mouse major histocompatibility complex (MHC) class II (11-5322; eBiosciences) and FITC-conjugated anti-mouse CD103 (11-1031; eBiosciences) antibodies, followed by staining with the appropriate fluorochrome-conjugated isotype control Ig. Flow cytometry was performed using a FACS Calibur flow cytometer (Becton-Dickinson, Mountain View, CA, USA).

### Statistical analysis

Data are presented as the mean ± standard deviation and statistical analysis was performed using SPSS 19.0 software (SPSS, Inc., Chicago, IL, USA). One-way analysis of variance followed by the Student-Newman-Keuls test was used for multiple comparisons, while the correlation coefficient was calculated using Spearman’s correlation analysis. P<0.05 was considered to indicate a statistically significant difference.

## Results

### NGF is upregulated in the lungs and serum of asthmatic mice

**Figure 1:**
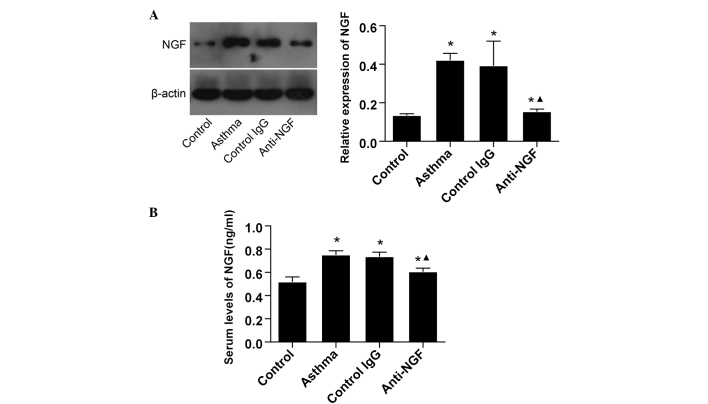
Ovalbumin sensitization and challenge results in increased NGF expression levels in mice. (A) Relative expression of NGF in the lungs was determined using western blot analysis (n=4). (B) NGF protein concentration in the serum was analyzed using an ELISA (n=7). Data are expressed as the mean ± standard deviation of individual groups. ^*^P<0.05, vs. control group; ^▲^P<0.05, vs. asthma and control IgG groups. NGF, nerve growth factor; IgG, immunoglobulin G.

Expression levels of NGF were analyzed in the lung tissues and serum to determine whether NGF production increased in asthmatic mice. Using western blot analysis and ELISA, NGF expression levels were shown to increase in the lungs and serum of asthmatic mice when compared with the control mice. As hypothesized, pretreatment with an anti-NGF monoclonal antibody decreased the NGF expression levels in the lung tissues and serum of asthmatic mice when compared with the mice in the control IgG and control groups (Fig. 1).

### NGF neutralization attenuates airway hyperreactivity (AHR), airway inflammation and the Th2 response in asthmatic mice

**Figure 2:**
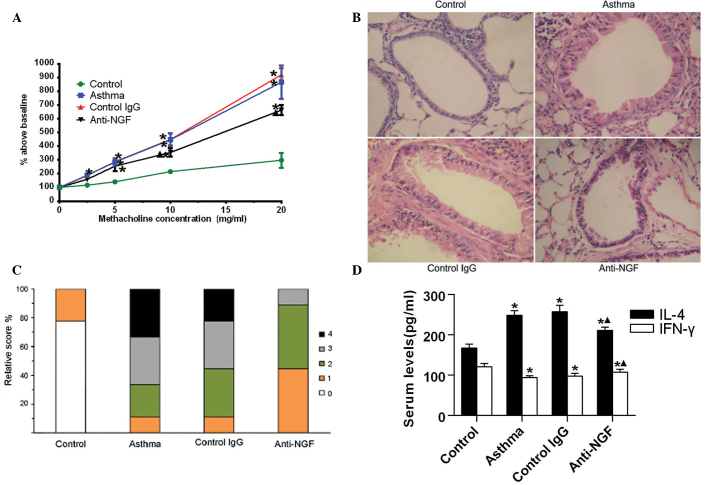
NGF neutralization attenuates airway hyperreactivity, airway inflammation and the T helper 2 response in asthmatic mice. Mice were sensitized via an intraperitoneal injection of 20 μg ovalbumin (OVA) and 1 mg aluminum hydroxide hydrate in 500 μl saline on days one and eight. From day 21, the mice were challenged with 5% OVA for five consecutive days. At 24 h following the last OVA challenge, mice were anesthetized and the airway responsiveness to methacholine was analyzed. (A) Changes in mice airway resistance (RL) in response to methacholine exposure. Data are expressed as a percentage above baseline (normal saline-induced RL) and presented as the mean ± standard deviation (n=7). (B) Representative photomicrographs of hematoxylin and eosin-stained lung sections from the mice (magnification, ×100). (C) Peribronchial inflammation scores of the mice; peribronchial inflammation of four mice from each group were analyzed and scored as follows: 0, normal; 1, few cells observed; 2, a ring of inflammatory cells one cell layer deep; 3, a ring of inflammatory cells 2–4 cells deep; and 4, a ring of inflammatory cells >4 cells deep. (D) Serum expression levels of IL-4 and IFN-γ in the mice were detected using an ELISA. Data are presented as the mean ± standard deviation (n=7). ^*^P<0.05, vs. control group; ^▲^P<0.05, vs. asthma and control IgG groups. NGF, nerve growth factor; IgG, immunoglobulin G; IFN, interferon; IL, interleukin.

As shown in Fig. 2A, at methacholine concentrations of ≥2.5 mg/ml, RL was found to significantly increase with increasing methacholine concentrations in the asthma group, as compared with the control group (P<0.05). Treatment with control IgG did not exhibit a marked effect on methacholine-induced hyperreactivity, with the RL in the control IgG-treated mice not significantly different when compared with the asthmatic mice. By contrast, treatment with anti-NGF antibodies significantly reduced the RL when compared with the asthma group; however, a number of RL values in the anti-NGF group mice remained significantly higher compared with those in the control group. These results revealed that anti-NGF treatment inhibits allergen-induced AHR in mice.

Histological examination of the lung tissue sections revealed that while the peribronchiolar infiltration of inflammatory cells was almost absent in the control mice, numerous inflammatory infiltrates were observed around the bronchial walls of the mice in the asthma and control IgG groups. By contrast, the content of inflammatory infiltrates and bronchial damage were reduced in the lungs of mice in the anti-NGF group (Fig. 2B). Quantitative analysis confirmed that the degree of peribronchial inflammation in the anti-NGF group decreased significantly compared with the asthma and control IgG groups (P<0.05; Fig. 2C). These observations indicated that pretreatment with anti-NGF antibodies significantly reduced the airway inflammatory response in sensitized mice when administered prior to the allergen challenge.

In addition, the serum expression levels of IL-4, as detected by ELISA, in the asthma and control-IgG groups were significantly higher when compared with the control group. However, the levels of IFN-γ were lower compared with those in the control group. By contrast, levels of IL-4 were reduced significantly, but levels of IFN-γ increased markedly, in the mice of the anti-NGF group when compared with those in the asthma and control-IgG groups (Fig. 2D). Therefore, these results indicated that NGF neutralization attenuates the Th2 response and strengthens the Th1 response in asthmatic mice.

### NGF neutralization inhibits the maturation of lung DCs in asthmatic mice

**Figure 3:**
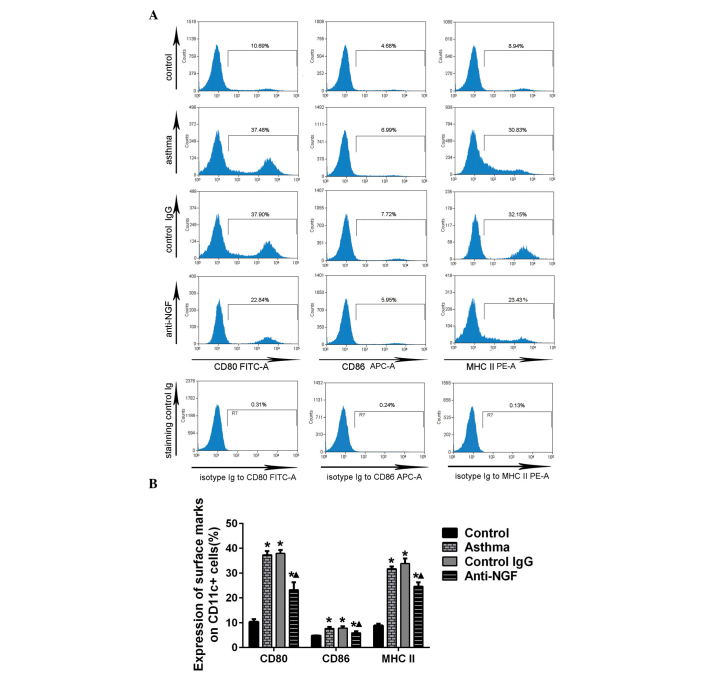
NGF neutralization inhibits the maturation of lung DCs in asthmatic mice. (A) Expression levels of CD80, CD86 and MHC class II in lung DCs were determined using flow cytometry. Representative data from one of the four replicate experiments are shown. (B) Histograms of compiled data. Data are expressed as the mean ± standard deviation (n=4).^*^P<0.05, vs. control group; ^▲^P<0.05, vs. asthma and control IgG groups. NGF, nerve growth factor; DCs, dendritic cells; MHC, major histocompatibility complex; IgG, immunoglobulin.

CD80, CD86 and MHC class II are surface markers that are highly expressed in mature DCs. The expression levels of CD80, CD86 and MHC class II on the lung DCs were found to be upregulated in the mice in the asthma and IgG control groups, as compared with the control group. A marked downregulation in the expression levels of CD80, CD86 and MHC class II was observed in the lung DCs of the mice in the anti-NGF group when compared with the mice in the asthma and control IgG groups (Fig. 3).

### NGF neutralization reduces the percentage of CD103^+^ lung DCs in asthmatic mice

**Figure 4:**
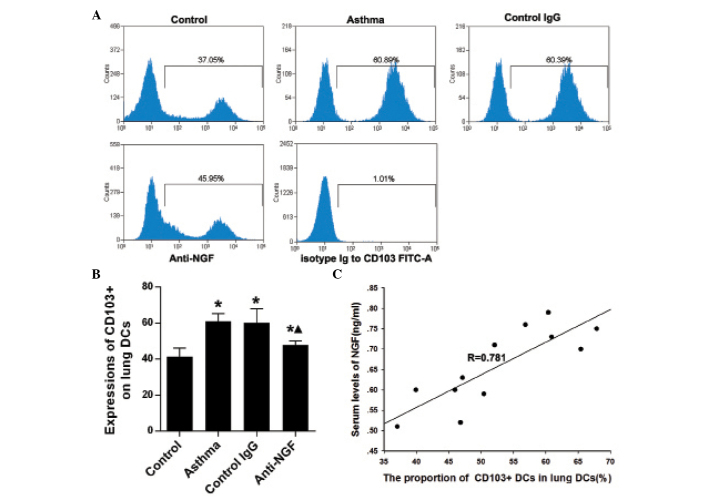
NGF neutralization reduces the proportion of lung CD103^+^ DCs in asthmatic mice. (A) Expression levels of CD103 in the lung DCs were determined using flow cytometry. Representative data from one of the three replicate experiments are shown. (B) Histograms of compiled data. Data are expressed as the mean ± standard deviation (n=3). (C) Correlation between the proportion of lung CD103^+^ DCs and the expression levels of NGF in the serum of the various groups of mice (n=12; R=0.781; P<0.01). ^*^P<0.05, vs. control group; ^▲^P<0.05, vs. asthma and control IgG groups. NGF, nerve growth factor; IgG, immunoglobulin; DCs, dendritic cells; CD, cluster of differentiation.

Compared with the control mice, the percentage of CD103^+^ lung DCs was significantly increased in the mice in the asthma and control IgG groups (P<0.05), as demonstrated by flow cytometric analysis. Anti-NGF treatment decreased the percentage of lung DCs with a CD103^+^ phenotype in asthmatic mice (Fig. 4A and B). Furthermore, as shown in Fig. 4C, the ratio of lung CD103^+^ DCs to lung DCs exhibited a positive correlation with the expression levels of NGF in the plasma of the mice. These results indicated that elevated levels of NGF may contribute to the increased number of lung CD103^+^ DCs in asthmatic mice.

## Discussion

Asthma is an airway inflammatory disease with an underlying Th2 cell-mediated inflammatory response in the airways (18). Increased levels of NGF expression are observed in patients with asthma following bronchial provocation with an allergen, and are associated with the severity of the inflammatory process and disease (19–21). Furthermore, a previous study demonstrated that NGF may exacerbate allergic lung inflammation in an animal model of asthma (22). However, the underlying mechanisms are yet to be fully elucidated. The observations of the present study provide new evidence indicating that NGF exacerbates lung inflammation by promoting lung DC maturation and polarization towards a Th2-stimulating phenotype in a mouse model of asthma.

In accordance with previous studies, the present study found that OVA-sensitized and challenged mice developed histopathological and biochemical features of asthma, including the infiltration of inflammatory cells in the airway, increased thickness of the basement membrane, increased airway responsiveness to methacholine and increased expression of NGF in the lungs and serum (22,23). In addition, anti-NGF treatment was shown to markedly reduce the levels of IL-4 and significantly increase the levels of IFN-γ in the serum of the mice. These results are consistent with previous observations (6,7,22), indicating the possible involvement of NGF in the development of the Th2 immune response. However, the mechanisms underlying NGF exacerbation of the Th2 inflammatory response in the airway remain unclear.

A novel observation of the present study was that NGF neutralization inhibits the maturation of lung DCs in asthmatic mice. Recently, an *in vivo* study (7) demonstrated that anti-NGF treatment significantly reduced allergic airway inflammation, upregulated the expression levels of IFN-γ and IL-10 and increased the number of Th1 and T regulatory cells; however, downregulated IL-4 and tumor necrosis factor (TNF)-α expression and reduced the number of Th2 and Th17 cells in a mouse model of asthma. These results indicated that NGF exacerbates allergic airway inflammation by modulating T cell responses (7). By functioning as a potent antigen-presenting cell (APC), DCs play an important role in initiating and maintaining T cell responses. Following allergen capture and processing, DCs mature, migrate to the T cell area in the draining mediastinal lymph node (LN) and induce a T cell response in the draining LN (24). During this process, DCs acquire a mature phenotype, where the expression levels of costimulatory molecules necessary for optimal naive T cell activation are upregulated, and acquire the capacity to stimulate an effector response (24). However, there is limited knowledge and research with regard to the role of NGF in the maturation and differentiation of DCs. In the present study, the expression levels of CD80, CD86 and MHC II were shown to be upregulated in the lung DCs of asthmatic mice when compared with the DCs in the control mice that were administered a vehicle only. Treatment with anti-NGF antibodies reduced OVA-stimulated CD80, CD86 and MHC class II expression in the DCs. Therefore, these results indicate that NGF may have an important role in promoting the maturation of lung DCs in asthmatic mice. However, the present study investigated the maturation of lung DCs *in vivo* only; thus, further *in vitro* investigations are required. Furthermore, results from previous *in vitro* experiments investigating whether NGF promotes the maturation of DCs are controversial (25,26). Noga *et al* (25) reported that following stimulation with NGF, no significant upregulation of CD80 and CD86 was observed in human monocyte-derived DCs (MoDCs). In addition, in allogeneic leucocyte reactions, MoDCs stimulated with NGF were unable to induce massive T-cell proliferation. By contrast, Jiang *et al* (26) demonstrated that NGF markedly promoted lipopolysaccharide (LPS)-induced expression of CD80, CD86 and the proinflammatory cytokines, IL-1, IL-6 and TNF-α, and the T cell-stimulating capacity of MoDCs, indicating that NGF may promote LPS-induced DC maturation (26). In addition, Braun *et al* (6) hypothesized that the Th2 response is not initiated under the influence of NGF, but instead an existing Th2 immune response is augmented by NGF. Therefore, further *in vivo* study is required to verify whether NGF may promote lung DC maturation in the background of allergic asthma. The results of the present study further the understanding of the mechanisms underlying NGF augmenting the Th2 response in asthma. IFN-γ is known to be a potent inducer of CD80, CD86 and MHC II on DCs (27). However, decreased expression levels of CD80, CD86 and MHC class II were observed in the lung DCs of the mice in the anti-NGF group, as compared with those in the asthma and control-IgG groups, despite increased serum levels of IFN-γ. One possible explanation is that a variety of factors determine the maturation of DCs, and IFN-γ is just one of these factors. Anti-NGF treatment may result in the decrease of other promoting factors or the increase of inhibiting factors in asthmatic mice; thus, leading to this seemingly contradictory phenomenon. However, further investigations are required to confirm this hypothesis.

An additional important observation of the present study was that NGF neutralization reduced the percentage of lung DCs with a CD103^+^ phenotype in asthmatic mice. Nakano *et al* (28) reported that lung CD103^+^ DCs may prime Th2 differentiation to inhaled allergens. Furthermore, mice lacking CD103^+^ DCs have been shown to produce markedly reduced allergic responses to clinically relevant allergens, including cockroach antigens and house dust extracts. Therefore, these results indicate that lung CD103^+^ DCs play a significant role in priming the Th2 response to inhaled antigens (28). In the present study, anti-NGF treatment prior to an OVA challenge was found to significantly reduce the number of lung CD103^+^ DCs in asthmatic mice, indicating that an NGF-induced Th2 response may be associated with an increased number of lung CD103^+^ DCs in asthmatic mice.

In conclusion, the results of the present study provide an explanation for the NGF promoting activation of lung DCs, which undergo maturation and polarization towards a Th2-stimulating phenotype, inducing a Th2 response in asthma. Therefore, downregulating NGF levels may benefit patients with allergic asthma. However, only limited conclusions can be derived from the present results and further studies are required to confirm these observations.
